# Health-Related Quality of Life After Breast Reconstruction: Comparing Outcomes Between Reconstruction Techniques Using the BREAST-Q

**DOI:** 10.1007/s00268-022-06677-9

**Published:** 2022-07-21

**Authors:** Charlotta Kuhlefelt, Pauliina Homsy, Jussi P. Repo, Tiina Jahkola, Susanna Kauhanen

**Affiliations:** 1 Division of Musculoskeletal and Plastic Surgery, Department of Plastic Surgery, University of Helsinki and Helsinki University Hospital, Park Hospital, HUS, Stenbäckinkatu 11, PB 281, 00029 Helsinki, Finland; 2 Unit of Musculoskeletal Diseases, Department of Orthopedics and Traumatology, Tampere University Hospital and University of Tampere, PB 2000, 33521 Tampere, Finland

## Abstract

**Background:**

Reconstruction of the breast following mastectomy can improve patients’ health-related quality of life (HRQL). We aimed to assess HRQL in women after mastectomy and breast reconstruction and to identify differences in HRQL related to the reconstruction method used.

**Methods:**

A cross-sectional study was performed on patients who had undergone breast reconstruction in Helsinki University Hospital between 08/2017 and 7/2019. The postoperative HRQL was assessed using the BREAST-Q (2.0) Reconstruction Module. The results were compared between patients with different reconstruction methods using the Kruskal–Wallis test.

**Results:**

A total of 146 patients were identified. Microvascular flaps (*n* = 77) were the most common method for primary breast reconstruction, followed by latissimus dorsi (LD) flaps (*n* = 45), fat grafting (*n* = 18) and implant reconstruction (*n* = 6). The satisfaction with breasts was high in all groups (median 61, IQR 49–71). The physical well-being of the chest was high regardless of the reconstructive method (median 100, IQR 80–100). However, women with fat grafting reported more adverse effects of radiation (median 17, IQR 14–17 vs. 18, IQR 17–18 for other groups, *p* = 0.02). Donor site morbidity was low, and patients reported high satisfaction with the back (median 66/100, IQR57-90) and abdomen (median 9/12, IQR 8–10), and physical well-being of the back (median 61/100, IQR 53–70) and abdomen (median 65/100, IQR 60–86).

**Conclusions:**

The patient-reported HRQL after breast reconstruction is high. Most women report being satisfied with the reconstruction, irrespective of the reconstruction method used. The reconstruction method can thus be chosen individually in cooperation between the patient and the surgeon.

**Supplementary Information:**

The online version contains supplementary material available at 10.1007/s00268-022-06677-9.

## Introduction

Surgery is a significant part of breast cancer treatment. Survival rates are high, accentuating patient satisfaction and health-related quality of life (HRQL) [1]. Breast reconstruction after mastectomy improves satisfaction, and women with reconstructed breast have been shown to score higher in several BREAST-Q scales compared to women with mastectomy alone [2–5].

Increased knowledge of reconstruction techniques and factors affecting surgical results has led to a more patient-centered approach when choosing the reconstructive method. These factors include prior radiation therapy to the donor area, donor adipose tissue composition, and microvascular anatomy [6]. Patient-reported outcome measures (PROMs) can be used to assess surgical outcomes and HRQL [1, 7].

Although multiple studies have demonstrated the positive effect of breast reconstruction, these studies often use varying PROMs, focus on a specific reconstruction method or compare autologous and implant-based reconstruction [2, 5, 8]. To our knowledge, no large number of previous studies have compared multiple reconstruction methods and their impact on HRQL using a breast-specific PROM [9].

The BREAST-Q is a psychometrically developed, breast-specific PROM for evaluating patient-reported HRQL and outcomes of breast surgery [10]. Since its development, the BREAST-Q has been translated into more than 30 languages and is widely used globally [11]. It has recently been translated to and validated in Finnish [12]. The BREAST-Q Reconstruction Module evaluates HRQL and satisfaction in patients who are about to, or have undergone, reconstructive surgery [13].

This study aimed to assess HRQL in women with breast reconstruction and evaluate possible differences in the BREAST-Q scores between reconstruction groups, previously unreported in a Finnish population. The setting of reconstruction methods is unique, as most of the patients had undergone autologous reconstruction. Furthermore, our cohort consisted of a large number of reconstructions with either latissimus dorsi (LD) flaps or free fat grafting.

## Patients and methods

### Methods

We performed a cross-sectional study on patients who had undergone breast reconstruction between 08/2017 and 7/2019 in Helsinki University Hospital, Department of Plastic Surgery. The patients were identified using operating theatre logs. A questionnaire package including the BREAST-Q questionnaire, a background information form, information on the study, a consent form, and a pre-paid return envelope was sent to the patients. The same questionnaire package was sent a second time if no answer was received.

The indication for mastectomy was either cancer (*n* = 115, 79%), or the presence of a cancer-associated gene (*n* = 20, 14%). Twelve patients (8.2%) had both cancer and a cancer-associated gene present. The decision to perform mastectomy was made between the patient and surgeon depending on the presence of cancer-associated genes, tumor size and other individual characteristics of the patient.

The patients’ medical records were viewed for health status, diagnosis, treatments given, and surgical therapy. The patients were divided into four groups depending on the primary reconstruction method. Only questions relevant to the operation the patients had undergone were analyzed.

The study protocol was approved by the Helsinki University Hospital ethics committee (HUS/2737/2017). Written consent was obtained from all participants.

### Study questionnaire

The BREAST-Q Reconstruction Module (version 2.0) was used to assess the patients’ HRQL. We used 14 postoperative scales for the study: Psychosocial Well-being, Sexual Well-being, Satisfaction with Breasts, Physical Well-being: Chest, Physical Well-being: Abdomen, Satisfaction with Abdomen, Satisfaction with Nipple Reconstruction, Satisfaction with Back, Physical Well-being: Back and Shoulder, Adverse Effects of Radiation, Satisfaction with Information, Satisfaction with Surgeon, Satisfaction with Medical Team and Satisfaction with Office Staff [14].

### Statistical analysis

Total scores for the BREAST-Q were rescaled to 0–100 with 0 indicating the worst and 100 the best outcome using the nonlinear Rasch transformation method [15]. Missing values on the scales were replaced with the mean score of the other items if less than 50% of the values were missing. Patients with more than 50% missing values on a scale were excluded from further analysis on that given scale. The scales for satisfaction with the abdomen, satisfaction with the nipple reconstruction and adverse effects of radiation were not rescaled. In these scales the scores on the individual answers were directly converted to the total score on that scale. This is in line with the instructions of the original authors of the BREAST-Q (2.0).

The results presented are given as the median, range, and interquartile range (IQR, 25th and 75th percentiles), if not stated otherwise. The Kruskal–Wallis test was used for groupwise comparison. Post hoc analysis was conducted using the Mann–Whitney U test with Bonferroni correction. All statistical tests were two-tailed and *p* values < 0.05 were considered statistically significant.

Statistical analysis was conducted by using IBM SPSS version 27 statistical software [16].

## Results

Of 338 patients identified, 146 patients (43%) participated in this study. The median age of the patients was 57 years (range 30–78, IQR 52–63). The median time from the first breast reconstruction until answering the BREAST-Q was 28 months (range 10–174, IQR 21–35,), and the median time from the last breast reconstruction procedure until answering the BREAST-Q was 16 months (range 0.2–38, IQR 10–21). Participant characteristics are shown in Tables 1 and 2.

**Table 1:** General characteristics of the study cohort

Variable	Median (IQR), range
Age (years)	57 (52–63), 30–78
Time from first reconstructive surgery to answering the BREAST-Q (months)	28 (21–35), 10–174
Time from last operation to answering the BREAST-Q (months)	16 (10–21), 0.2–38
BMI (kg/m^2^)	25 (23–28), 18–34

Variable	Group	*N* (%)
Active smoker	Yes	5 (3.4)
	No	139 (93)
	Missing	2 (1.4)
ASA-classification^a^	ASA I	74 (51)
	ASA II-III	72 (49)
Reason for surgery	Cancer	115 (79)
	Cancer-associated gene	20 (14)
	Both	11 (7.5)
Cancer-related gene	BRCA1	12 (8.2)
	BRCA2	11 (7.5)
	PALB2	3 (2.1)
	FANCM	2 (1.4)
	CHEK2	1 (0.7)
	Unknown	2 (1.4)
	None	115 (79)
Prior breast cancer	Other side	15 (10)
	Same side	6 (4.1)
	Both	2 (1.4)
	No	123 (84)

^a^*ASA I* a normal healthy patient, *ASA II* a patient with mild systemic disease, *ASA III* a patient with severe systemic disease

**Table 2:** Surgical descriptive of the study cohort

Variable	Group		*N* (%)
Type of cancer	DCIS^a^		23 (16)
	Ductal carcinoma		66 (45)
	LCIS^b^		2 (1.4)
	Lobular carcinoma		23 (16)
	Other or undetermined		12 (8.2)
	Not cancer		20 (14)
Bilateral surgery	Yes		81 (56)
	No		64 (45)
Contralateral surgery for aesthetic reasons	Reduction mammoplasty		20 (14)
	Mastopexy		9 (6.2)
	Mastectomy		1 (0.7)
	None		116 (71)
Time of reconstruction	Immediate		77 (53)
	Delayed		69 (47)
Primary reconstruction method	LD		45 (31)
		With implant	3 (6.7)
		Without implant	42 (93)
	Microvascular flap		77 (53)
		Abdominal flap	64 (83)
		Gracilis flap	13 (17)
	Fat graft		18 (12)
	Implant		6 (4.1)
Total no of reconstructive surgeries^c^	1		27 (18)
		LD	9 (20)
		Microvascular flap	17 (22)
		Implant	0 (0)
		Fat graft	1 (5.6)
	2-3^d^		85 (58)
		LD	25 (56)
		Microvascular flap	50 (65)
		Implant	2 (33)
		Fat graft	8 (44)
	4–5		24 (16)
		LD	10 (22)
		Microvascular flap	7 (9.1)
		Implant	1 (17)
		Fat graft	6 (33)
	5 or more		10 (6.8)
		LD	1 (2.2)
		Microvascular flap	3 (3.9)
		Implant	3 (50)
		Fat graft	3 (17)
Clavien-Dindo classification^e^	0		81 (55)
		LD	17 (38)
		Microvascular flap	42 (55)
		Implant	4 (67)
		Fat graft	18 (100)
	1		40 (27)
		LD	22 (49)
		Microvascular flap	17 (22)
		Implant	1 (17)
		Fat graft	0 (0)
	2		3 (2.1)
		LD	2 (4.4)
		Microvascular flap	1 (1.3)
		Implant	0 (0)
		Fat graft	0 (0)
	3a		2 (1.4)
		LD	1 (2.2)
		Microvascular flap	0 (0)
		Implant	1 (17)
		Fat graft	0 (0)
	3b		20 (14)
		LD	3 (6.7)
		Microvascular flap	17 (22)
		Implant	0 (0)
		Fat graft	0 (0)
Complications	Prolonged wound care		13 (8.9)
	Seroma		29 (20)
	Exploration surgery		2 (1.4)
	Bleeding		4 (2.7)
	Evacuation of hematoma		9 (6.2)
	Re-anastomosis		6 (4.1)
	Primary revision surgery		7 (4.8)
	Secondary revision surgery		11 (7.5)
	Scar revision		14 (9.6)
	Removal of the flap		2 (1.4)
	Other		2 (1.4)
	None		68 (47)
Reconstruction of the nipple	Yes		67 (46)
	No		79 (54)

^a^Ductal carcinoma in situ

^b^Lobular carcinoma in situ

^c^Excluding nipple reconstructions and surgery due to complications

^d^Six out of 66 patients had bilateral reconstructions and only one reconstruction surgery per breast

^e^Grade I: Any deviation from the normal postoperative course without the need for pharmacological treatment or surgical, endoscopic, and radiological interventions. Allowed therapeutic regimens are: drugs as antiemetics, antipyretics, analgetics, diuretics, electrolytes, and physiotherapy. This grade also includes wound infections opened at the bedside. Grade II: Requiring pharmacological treatment with drugs other than such allowed for grade I complications. Grade III: Requiring surgical, endoscopic or radiological intervention. IIIa: Intervention not under general anesthesia. IIIb: Intervention under general anesthesia

Of all patients, 119 (82%) had undergone more than one reconstructive surgery, including nipple reconstructions and reoperations due to postoperative complications. Sixty-eight patients (47%) experienced no postoperative complications. The most common complications included seromas (*n* = 29, 20%) and prolonged wound care (*n* = 13, 9%). Seven patients (4.8%) required primary revision surgery, and two patients (1.4%) suffered flap loss. (Table 2).

Microvascular flaps were used for 77 patients (53%), including abdominal flaps (*n* = 64, 83%) and gracilis flaps (*n* = 13, 17%). Abdominal flaps included the deep inferior epigastric perforator flap, DIEP (*n* = 45, 70%), the transverse abdominal rectus muscle flap, TRAM (*n* = 13, 20%), the superficial inferior epigastric artery flap, SIEA (*n* = 3, 5%), and the lumbar artery perforator flap, LAP (*n* = 3, 5%). LD flaps, with or without implant or fat enhancement, were used for 45 patients (31%). Free fat grafts were used for 18 patients (12%). Implants were used for six patients (4%) The satisfaction with implant scale was excluded from further analysis due to low response rate (*n* = 1).

Of the reconstructions performed, 77 (53%) was immediate and 69 (47%) delayed. Radiotherapy was given to 76 patients (52%) postoperatively. Twenty patients (26%) with immediate reconstruction received postoperative radiotherapy. If radiotherapy was given prior to reconstruction, delayed reconstruction was performed 12 months after radiotherapy at the earliest. Forty-three patients (62%) who underwent delayed reconstruction had received radiotherapy prior to the reconstruction. On these patients, reconstruction was performed with microvascular flaps (*n* = 27, 63%), fat grafts (*n* = 10, 23%) and LD flaps (*n* = 6, 14%).

### Psychosocial and sexual well-being

**Fig. 1:**
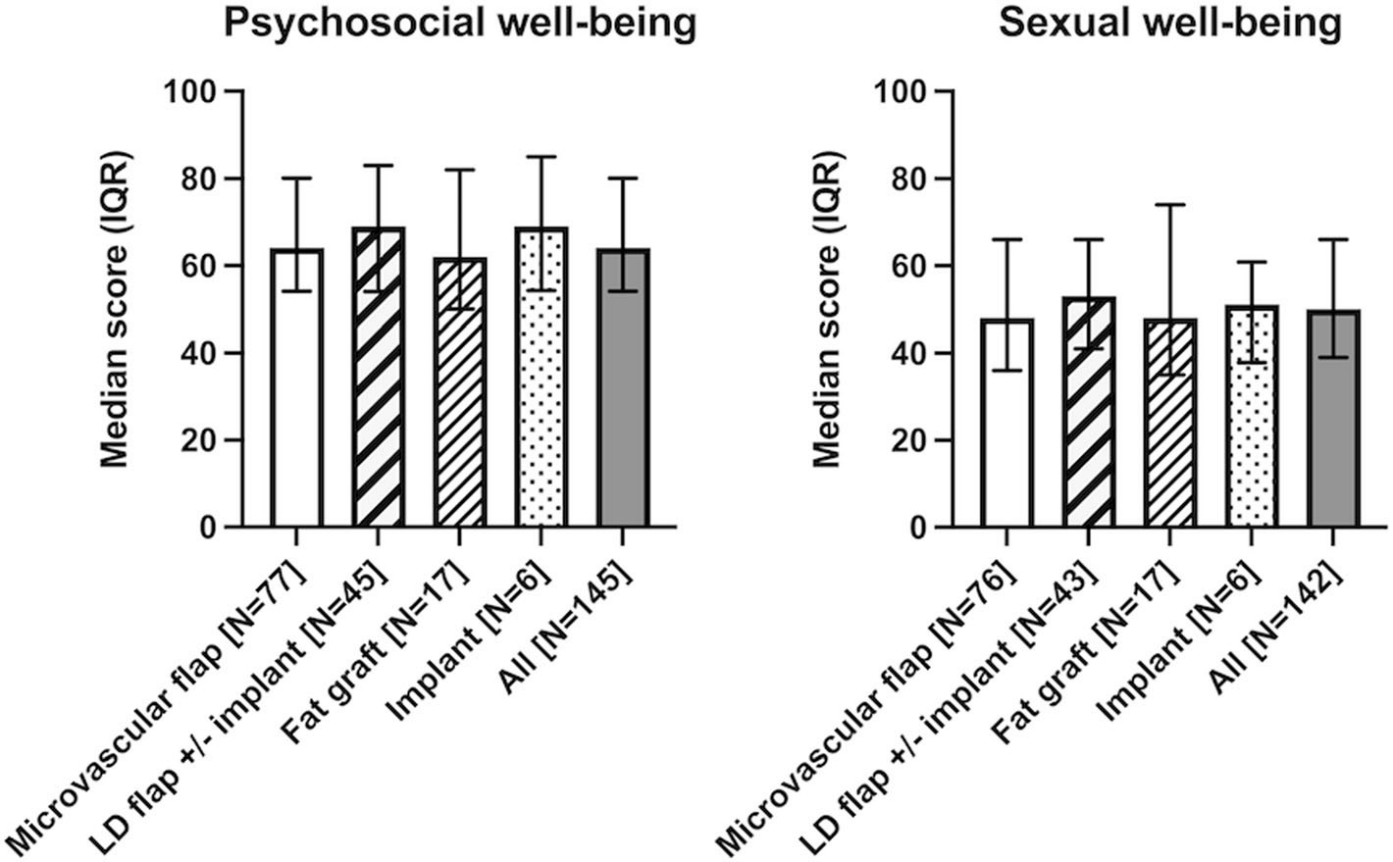
BREAST-Q scores for psychosocial and sexual well-being in women 2.3 (0.8–14.3) years after breast reconstruction

Psychosocial well-being was similar in all reconstruction groups (*p* = 0.78). The median value for all patients was 64 (range 24–100, IQR 54–80). The scores for sexual well-being had an overall median of 50 (range 0–100, IQR 39–66). No significant difference between reconstruction methods was detected (*p* = 0.77). (Fig. 1, Supplemental Table 1a).

### Satisfaction with breast and physical well-being: chest

**Fig. 2:**
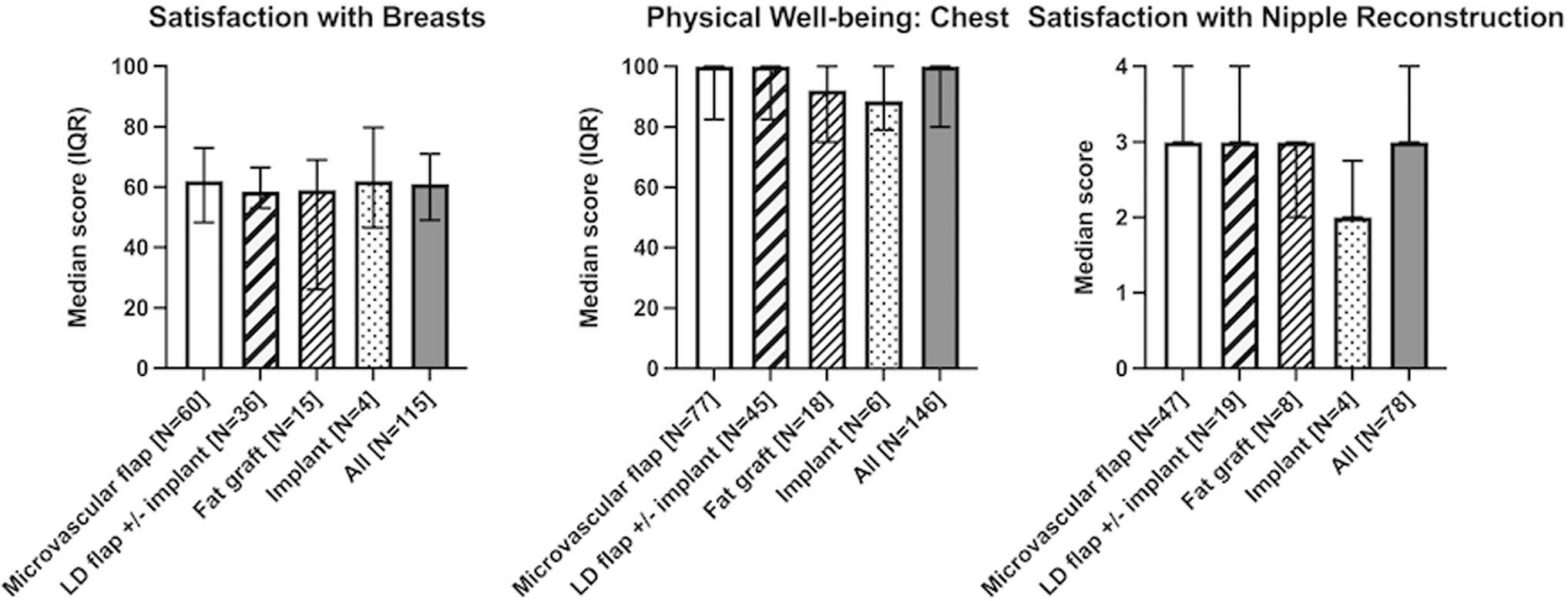
BREAST-Q scores for satisfaction with breasts, physical well-being: chest and satisfaction with nipple reconstruction in women 2.3 (0.8–14.3) years after breast reconstruction

The median score for satisfaction with breast was 61 (range 0–100, IQR 49–71). No significant difference was detected between patients who had undergone breast reconstruction with either microvascular flaps (*n* = 60, median 62, IQR 48–73), LD flaps (*n* = 36, median 59, IQR 53–67), fat grafts (*n* = 15, median 59, IQR 26–69) or implants (*n* = 4, median 62, IQR 47–80), *p* = 0.47. (Fig. 2, Supplemental Table 1b) The physical well-being of the chest was high (median100, range 50–100, IQR 80–100). No significant difference was detected among the reconstruction groups (*p* = 0.56), and the results were similar in patients with microvascular flaps (*n* = 77, median 100, IQR 83–100), LD flaps (*n* = 45, median 100, IQR 83–100), fat grafts (*n* = 18, median 92, IQR 75–100) and implants (*n* = 6, median 89, IQR 79–100). (Fig. 2, Supplemental Table 1b).

### Satisfaction with nipple reconstruction

Seventy-eight patients (53%) answered the scale for the satisfaction with nipple reconstruction. The median, on a 1–4-point scale, was 3 (range 1–4, IQR 3–4). There was a significant difference in satisfaction between the reconstruction groups (*p* = 0.04). The patients with microvascular flaps demonstrated the highest satisfaction. In groupwise comparison, no significant difference was detected between microvascular flaps or LD flaps. The implant group (*n* = 4, median 2, range 2–3, IQR 2–3) reported lower satisfaction than both patients with LD flaps (*n* = 19, median 3, range 1–4, IQR 3–4; *p* = 0.03) and microvascular flaps (*n* = 47, median 3, range 1–4, IQR 3–4; *p* = 0.02). However, only four patients (67%) with implant reconstruction answered this scale. (Fig. 2, Supplemental Table 1b).

### Satisfaction with back and physical well-being: back and shoulder

Forty-one of the 45 patients (91%) with LD flaps answered the Satisfaction with Back scale. The median value was 66 (range 50–100, IQR 57–90). The response rate for the physical well-being of the back and shoulder scale was 93%. The median value was 61 (range 35–100, IQR 53–70). (Supplemental Table 1e).

### Satisfaction with abdomen and physical well-being: abdomen

Satisfaction with the abdomen and physical well-being of the abdomen were analyzed for patients who had underwent abdominal flap reconstruction (*n* = 64). The response rate for the Satisfaction with Abdomen scale was 91%. On a 3–12-point scale, the median score was 9 (range 3–12, IQR 8–10). The physical well-being of the abdomen had a median score of 60 (range 47–100, IQR 60–86). The response rate for this scale was 94%. (Supplemental Table 1e).

### Adverse effects of radiation

**Fig. 3:**
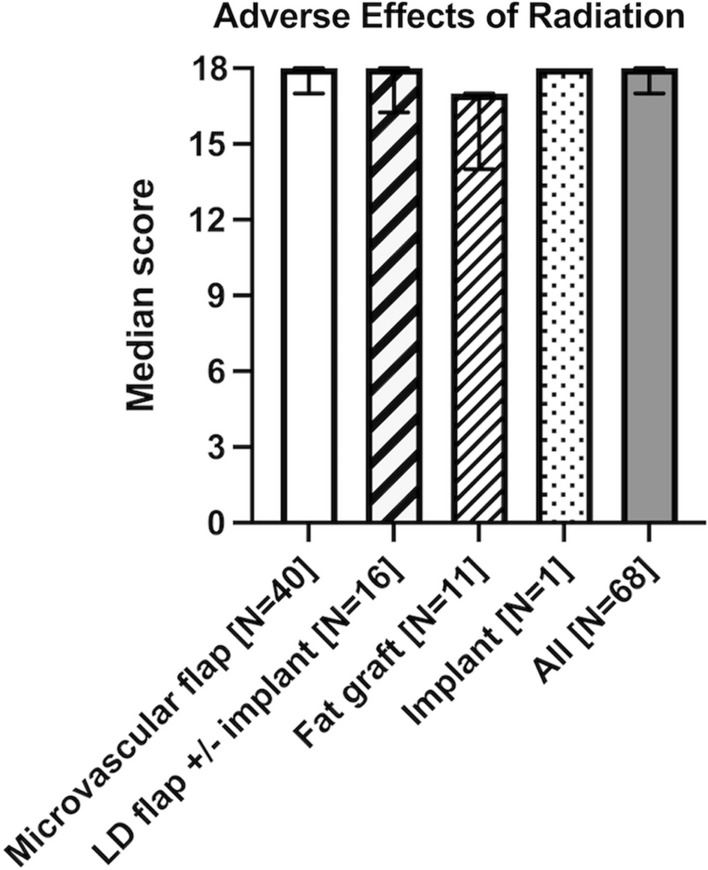
BREAST-Q scores for adverse effects of radiation in women 2.3 (0.8–14.3) years after breast reconstruction

Seventy-six patients (52%) had received radiation therapy to the chest. Sixty-eight patients answered the scale on the adverse effects of radiation, which constituted 89% of all women with prior radiation therapy and 47% of all patients. This scale measures possible physical changes of the irradiated skin. These include dryness, lack of pliability, increased sensitivity, increased thickness, soreness and scarring of the skin. Low scores in this scale are associated with adverse effects of radiation. There was a significant difference between the reconstruction groups (*p* = 0.02). The fat graft group reported the lowest scores, with a median score of 17 (*n* = 11, range 12–18, IQR 14–17). This group reported lower scores than patients with microvascular flaps (*n* = 40, median 18, range 10–18, IQR 17–18; *p* = 0.002) and LD flaps (*n* = 16, median 18, range 14–18, IQR 16–18; *p* = 0.02). (Fig. 3, Supplemental Table 1c) The overall median was 18 (range 10–19, IQR 17–18). One patient with implant reconstruction answered this scale and the implant group was thus excluded from the analysis.

### Satisfaction with care

**Fig. 4:**
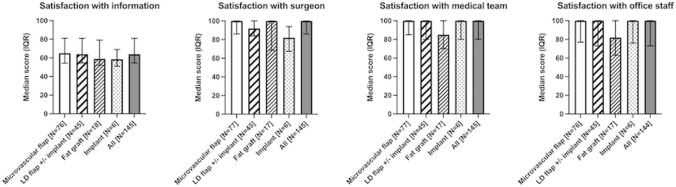
BREAST-Q scores for satisfaction with information, satisfaction with surgeon, satisfaction with medical team and satisfaction with office staff in women 2.3 (0.8–14.3) years after breast reconstruction

Scores for the scales regarding the satisfaction with the care were high (Fig. 4, Supplemental Table 1d). No significant difference was detected between the reconstruction groups in satisfaction with information (median 64, range 32–100, IQR 55–81, *p* = 0.64), satisfaction with surgeon (median 100, range 22–100, IQR 86–100, *p* = 0.23), satisfaction with medical team (median 100, range 0–100, IQR 80–100, *p* = 0.35) or satisfaction with office staff (median 100, range 0–100, IQR 73–100, *p* = 0.20).

## Discussion

Reconstruction of the breast after mastectomy often improves the HRQL [2, 3, 17, 18]. Autologous reconstruction is associated with higher satisfaction compared to implant-based reconstruction [19]. Our study cohort reported high scores in the BREAST-Q regardless of reconstruction method. The patients with LD or abdominal flaps reported high satisfaction with the back and abdomen.

Breast satisfaction was high in our study population. The median, 61, was in line with prior studies assessing the HRQL in women breast reconstruction (mean range 58–71) [2, 8]. Similar values have been observed in healthy control groups [3]. Interestingly, the score is above the proposed normative mean score, 58, derived from answers of women with no prior history of breast cancer or breast surgery [14].

The median for the physical well-being of the chest was 100, being higher than the estimated normative mean score for this scale, 93 [14]. Autologous reconstruction is associated with greater satisfaction compared to implant-based reconstruction [8]. In our study, the implant group reported lower physical well-being of the chest, although no significant difference was detected. However, with only six patients with implant-based reconstruction, no informative comparison could be made between autologous and implant-based reconstruction.

Microvascular flaps formed the largest group in our study (*n* = 77, 53%). These patients reported the highest satisfaction with the breast and physical well-being of the chest, although not statistically significant. The satisfaction with the abdomen and the physical well-being of the abdomen were high. These results are similar to other studies measuring BREAST-Q scores after breast reconstruction using abdominal flaps [20, 21]. However, the physical well-being of the abdomen falls short of the normative mean score for the scale, 78 [14].

Forty-five (31%) patients had undergone LD flap reconstruction, reflecting the popularity of this reconstruction method in Finland and Scandinavia [22, 23]. It is considered favorable for women with small breasts or excess fat tissue in the back [24]. The LD flap has been associated with high patient satisfaction, positive aesthetic results and low complication rates [25, 26]. However, several studies have reported impaired functionality and long-term discomfort after reconstruction [27]. In our cohort, the LD group reported relatively high satisfaction with the breast and the back. These patients scored the highest in psychosocial and sexual well-being as well as the physical well-being of the chest, although no statistically significant difference was detected. Similar BREAST-Q scores have been reported in prior studies [26].

Breast reconstruction with free fat transfer is an increasingly popular option in our patient group [28]. Although previously used for filling tissue defects or as an additional reconstructive method, serial fat grafting is feasible as the sole reconstructive method [29, 30]. Fat grafting is associated with low complication rates and satisfactory cosmetic outcomes [31]. In our study, 18 patients (12%) underwent primary reconstruction using fat grafting. These patients reported relatively high satisfaction with the breast (median 59) and physical well-being of the chest (median 92). Interestingly, the fat graft group reported more adverse effects of radiation than patients with microvascular and LD flaps. While fat transfer has been suggested to reduce the skin effects of irradiation damage [32], this may reflect the presence of only the original, irradiated skin envelope in the breast. Additionally, patients with fat grafts are often informed about the possible effects of radiotherapy on graft retention.

Our study cohort reported high satisfaction with the nipple reconstruction. Nipple reconstruction is associated with improved satisfaction, as well as psychosocial and sexual well-being [33–35]. Nipple reconstruction is routinely offered to all our patients, and 78 (53%) of the study participants had a reconstructed nipple. Patients with autologous flaps were more satisfied with the nipple reconstruction than patients with implant reconstructions. However, our results might have been affected by the low number of patients (*n* = 4) with implant-based reconstruction who answered this scale.

Total donor site morbidity in our study was low, and physical well-being of the back and shoulder (median 61) and abdomen (median 9) were high. Our small implant group complicated the comparison between implant-based and autologous reconstruction. However, low donor site morbidity, combined with high overall satisfaction independent of the reconstruction method, could be considered to favor autologous reconstruction methods. This is especially so as autologous reconstructions have been associated with a greater HRQL and patient-reported satisfaction [2, 8, 19].

Study limitations include the cross-sectional design with a lack of a known preoperative HRQL and control groups. The time from surgery was up to four years, therefore enough to surpass the initial postoperative decrease in HRQL [36]. Although our cohort included all patients undergoing breast reconstruction in this hospital in a three-year period, selection bias is possible due to the high number of non-responders. However, the response rate is similar to the average response rate of mail surveys [37]. Further, the cohort included patients of a wide age range and multiple reconstructive procedures. Most of the reconstructions were autologous, and we had a large group of patients with free fat transfer as primary reconstructive method. Even so, some of the reconstruction groups were too small to enable meaningful comparisons between methods. This also entailed that no comparison was possible between different microvascular reconstruction donor sites. Further, with only six patients with implant reconstructions, our study is likely to have been underpowered to demonstrate a potential difference in the HRQL between the implant-based and autologous reconstruction.

The low number of implant-based reconstructions in our study reflects the practice in our department, with implant reconstructions being performed in the Breast Cancer Unit instead of the Plastic Surgery Department. In contrast, our study included several reconstruction methods, and we had a large group of patients with reconstruction using fat grafts in the analysis, a patient group infrequently represented in other similar studies.

In conclusion, women with breast reconstructions reported high satisfaction with the breasts and few adverse effects, irrespective of the reconstruction method used. Therefore, the decision regarding the method of breast reconstruction can be made individually considering the patients’ wishes and individual characteristics.

## Supplementary Information

Below is the link to the electronic supplementary material.

Supplementary file1 (DOCX 20 kb)

## References

[1] Torre LA, Siegel RL, Ward EM (2016). Global cancer incidence and mortality rates and trends—an update. Cancer Epidemiol Biomark Prev.

[2] Eltahir Y, Werners LLCH, Dreise MM (2013). Quality-of-life outcomes between mastectomy alone and breast reconstruction: comparison of patient-reported BREAST-Q and other health-related quality-of-life measures. Plast Reconstr Surg.

[3] Howes BHL, Watson DI, Xu C (2016). Quality of life following total mastectomy with and without reconstruction versus breast-conserving surgery for breast cancer: a case-controlled cohort study. J Plast Reconstr Aesthet Surg.

[4] Zehra S, Doyle F, Barry M (2020). Health-related quality of life following breast reconstruction compared to total mastectomy and breast-conserving surgery among breast cancer survivors: a systematic review and meta-analysis. Breast Cancer.

[5] Rautalin M, Jahkola T, Roine RP (2021). Surgery and health-related quality of life—a prospective follow up study on breast cancer patients in Finland. Eur J Surg Oncol.

[6] Panchal H, Matros E (2017). Current trends in post-mastectomy breast reconstruction. Plast Reconstr Surg.

[7] Pusic AL, Chen CM, Cano S (2007). Measuring quality of life in cosmetic and reconstructive breast surgery: a systematic review of patient-reported outcomes instruments. Plast Reconstr Surg.

[8] Santosa KB, Qi J, Kim HM (2018). Long-term patient-reported outcomes in postmastectomy breast reconstruction. JAMA Surg.

[9] Jeevan R, Cromwell D, Browne J et al (2011) National mastectomy and breast reconstruction audit 2011. Fourth annual report

[10] Pusic AL, Klassen AF, Scott AM (2009). Development of a new patient-reported outcome measure for breast surgery: the BREAST-Q. Plast Reconstr Surg.

[11] BREAST-Q Version 2.0 © (2017) A guide for researchers and clinicians

[12] BREAST-Q|Breast Cancer—Q-Portfolio. Adaptable to your needs. https://qportfolio.org/breast-q/breast-cancer/. Accessed 23 Feb 2022

[13] Cohen WA, Mundy LR, Ballard TN (2016). The BREAST-Q in surgical research: a review of the literature 2009–2015. J Plast Reconstr Aesthet Surg.

[14] Mundy LR, Homa K, Klassen AF (2017). Breast cancer and reconstruction: normative data for interpreting the BREAST-Q. Plast Reconstr Surg.

[15] Cano S, Klassen A, Cano SJ et al (2011) From BREAST-Q © to Q-SCORE ©: using Rasch easurement to better capture breast surgery outcome. Joint International IMEKO TC1+ TC7+ TC13 Symposium, August 31st−September 2nd, 2011, Jena, Germany. urn:nbn:de:gbv:ilm1-2011imeko:2

[16] IBM Corp. Released 2020. IBM SPSS statistics for Windows, version 27.0. IBM Corp, Armonk

[17] Wang X, Zhu K, Ren L (2020). Quality of life and related risk factors after breast reconstruction in breast cancer patients. Gland Surg.

[18] Rautalin M, Jahkola T, Roine RP (2022). Breast reconstruction-prospective follow up on breast cancer patients’ health-related quality of life. World J Surg.

[19] Toyserkani NM, Jørgensen MG, Tabatabaeifar S (2020). Autologous versus implant-based breast reconstruction: a systematic review and meta-analysis of Breast-Q patient-reported outcomes. J Plast Reconstr Aesthet Surg.

[20] Liu T, Freijs C, Klein HJ (2018). Patients with abdominal-based free flap breast reconstruction a decade after surgery: a comprehensive long-term follow-up study. J Plast Reconstr Aesthet Surg.

[21] Boczar D, Huayllani MT, Forte AJ (2020). Microsurgical breast reconstruction in the obese patient using abdominal flaps: complication profile and patient satisfaction. Ann Plast Surg.

[22] Schantz PAM, Kauhanen MSC (2021). The versatile latissimus dorsi flap: old and reliable or outmoded—with or without an add on?. Ann Breast Surg.

[23] Eriksen C, Stark B (2009). The latissimus dorsi flap—still a valuable tool in breast reconstruction: report of 32 cases. Scand J Plast Reconstr Surg Hand Surg.

[24] Palve J, Luukkaala T, Kääriäinen M (2022). Comparison of different techniques in latissimus dorsi breast reconstruction. Ann Plast Surg.

[25] Mericli AF, Szpalski C, Schaverien M (2019). The latissimus dorsi myocutaneous flap is a safe and effective method of partial breast reconstruction in the setting of breast-conserving therapy. Plast Reconstr Surg.

[26] Wattoo G, Nayak S, Khan S (2021). Long-term outcomes of latissimus dorsi flap breast reconstructions: a single-centre observational cohort study with up to 12 years of follow up. J Plast Reconstr Aesthet Surg.

[27] Lee KT, Mun GH (2014). A systematic review of functional donor-site morbidity after latissimus dorsi muscle transfer. Plast Reconstr Surg.

[28] Kauhanen S, Höckerstedt A (2019). Full breast reconstruction with fat and how to recycle the “dog-ear”. Gland Surg.

[29] Khouri KS, Khouri RK (2019). The third postmastectomy reconstruction option-autologous fat transfer. JAMA Surg.

[30] Hoppe DL, Ueberreiter K, Surlemont Y (2013). Breast reconstruction de novo by water-jet assisted autologous fat grafting—a retrospective study. GMS German Med Sci.

[31] Khouri RK, Eisenmann-Klein M, Cardoso E et al (2015) Brava and autologous fat transfer is a safe and effective breast augmentation alternative: results of a 6-year, 81-patient, prospective multicenter study. In: Plastic surgery complete: the clinical masters of PRS—breast augmentation, pp 82–96.10.1097/PRS.0B013E31824A2DB6 10.1097/PRS.0b013e31824a2db6 22261565

[32] Rigotti G, Marchi A, Galiè M (2007). Clinical treatment of radiotherapy tissue damage by lipoaspirate transplant: a healing process mediated by adipose-derived adult stem cells. Plast Reconstr Surg.

[33] Egan KG, Cullom M, Nazir N (2021). Patient satisfaction increases with nipple reconstruction following autologous breast reconstruction. Plast Reconstr Surg.

[34] Momoh AO, Colakoglu S, de Blacam C (2012). The impact of nipple reconstruction on patient satisfaction in breast reconstruction. Ann Plast Surg.

[35] Bykowski MR, Emelife PI, Emelife NN (2017). Nipple–areola complex reconstruction improves psychosocial and sexual well-being in women treated for breast cancer. J Plast Reconstr Aesthet Surg.

[36] Shimozuma K, Ganz PA, Petersen L (1999). Quality of life in the first year after breast cancer surgery: rehabilitation needs and patterns of recovery. Breast Cancer Res Treat.

[37] Shih T-H, Fan X (2008) Comparing response rates from web and mail surveys: a meta-analysis.10.1177/1525822X08317085

