# Supplementary material for: Health-Related Quality of Life After Breast Reconstruction: Comparing Outcomes Between Reconstruction Techniques Using the BREAST-Q
Source: World J Surg. 2022 Jul 21;46(11):2695–705. doi: 10.1007/s00268-022-06677-9 (PMC9529680; doi:10.1007/s00268-022-06677-9)
Supplement: Supplementary file 1 — Supplementary file1 (DOCX 20 kb) [file 268_2022_6677_MOESM1_ESM.docx]

Supplemental Table 1a. Psychosocial well-being and Sexual well-being.

| **Scale** | **Reconstruction group** | **N (%)** | **Median**  **(25th/75th percentile)** | **Min** | **Max** | **P-value** |
| --- | --- | --- | --- | --- | --- | --- |
| **Psychosocial**  **well-being** | Microvascular flap | 77 (100) | 64 (54-80) | 24 | 100 | 0.78 |
|  | LD flap | 45 (100) | 69 (54-83) | 32 | 100 |  |
|  | Fat graft | 17 (94) | 62 (50-82) | 35 | 100 |  |
|  | Implant | 6 (100) | 69 (54-85) | 52 | 100 |  |
|  | Overall | 145 (99) | 64 (54-80) | 24 | 100 |  |
| **Sexual well-**  **being** | Microvascular flap | 76 (99) | 48 (36-66) | 0 | 100 | 0.77 |
|  | LD flap | 43 (96) | 53 (41-66) | 0 | 91 |  |
|  | Fat graft | 17 (94) | 48 (35-74) | 20 | 100 |  |
|  | Implant | 6 (100) | 51 (38-61) | 34 | 66 |  |
|  | Overall | 142 (97) | 50 (39-66) | 0 | 100 |  |

LD, latissimus dorsi; N, number of patients

Supplemental Table 1b. Satisfaction with breasts, Physical well-being: Chest and Satisfaction with nipple reconstruction.

| **Scale** | **Reconstruction group** | **N (%)** | **Median (25th/75th**  **percentile)** | **Min** | **Max** | **P-**  **value** |
| --- | --- | --- | --- | --- | --- | --- |
| **Satisfaction**  **with breast** | Microvascular flap | 60 (78) | 62 (48-73) | 0 | 100 | 0.47 |
|  | LD flap | 36 (80) | 59 (53-67) | 36 | 92 |  |
|  | Fat graft | 15 (83) | 59 (26-69) | 21 | 100 |  |
|  | Implant | 4 (67) | 62 (47-80) | 45 | 82 |  |
|  | Overall | 115 (79) | 61 (49-71) | 0 | 100 |  |
| **Physical well-**  **being: chest** | Microvascular flap | 77 (100) | 100 (83-100) | 55 | 100 | 0.56 |
|  | LD flap | 45 (100) | 100 (83-100) | 60 | 100 |  |
|  | Fat graft | 18 (100) | 92 (75-100) | 50 | 100 |  |
|  | Implant | 6 (100) | 89 (79-100) | 76 | 100 |  |
|  | Overall | 146 (100) | 100 (80-100) | 50 | 100 |  |
| **Satisfaction with nipple**  **reconstruction** | Microvascular flap | 47 (61) | 3 (3-4) | 1 | 4 | 0.04 |
|  | LD flap | 19 (42) | 3 (3-4) | 1 | 4 |  |
|  | Fat graft | 8 (44) | 3 (2-3) | 1 | 4 |  |
|  | Implant | 4 (67) | 2 (2-3) | 2 | 3 |  |
|  | Overall | 78 (53) | 3 (3-4) | 1 | 4 |  |

LD, latissimus dorsi; N, number of patients

Supplemental Table 1c. Adverse effects of radiation.

| **Scale** | **Reconstruction group** | **N (%)** | **Median**  **(25th/75th percentile)** | **Min** | **Max** | **P-value** |
| --- | --- | --- | --- | --- | --- | --- |
| **Adverse**  **effects of radiation** | Microvascular flap | 40 (52) | 18 (17-18) | 10 | 18 | 0.02 |
|  | LD flap | 16 (36) | 18 (16-18) | 14 | 18 |  |
|  | Fat graft | 11 (61) | 17 (14-17) | 12 | 18 |  |
|  | Implant | 1 (17) |  |  |  |  |
|  | Overall | 68 (47) | 18 (17-18) | 10 | 18 |  |

LD, latissimus dorsi; N, number of patients

Supplemental Table 1d. Satisfaction with care.

| **Scale** | **Reconstruction group** | **N (%)** | **Median**  **(25th/75th percentile)** | **Min** | **Max** | **P-value** |
| --- | --- | --- | --- | --- | --- | --- |
| **Satisfaction**  **with information** | Microvascular flap | 76 (99) | 65 (54-81) | 33 | 100 | 0.64 |
|  | LD flap | 45 (100) | 64 (55-81) | 32 | 100 |  |
|  | Fat graft | 18 (100) | 59 (52-79) | 35 | 100 |  |
|  | Implant | 6 (100) | 59 (51-69) | 39 | 81 |  |
|  | Overall | 145 (99) | 64 (54-81) | 32 | 100 |  |
| **Satisfaction**  **with surgeon** | Microvascular flap | 77 (100) | 100 (86-100) | 29 | 100 | 0.23 |
|  | LD flap | 45 (100) | 92 (84-100) | 22 | 100 |  |
|  | Fat graft | 17 (94) | 100 (69-100) | 38 | 100 |  |
|  | Implant | 6 (100) | 82 (68-94) | 45 | 100 |  |
|  | Overall | 145 (99) | 100 (86-100) | 22 | 100 |  |
| **Satisfaction**  **with medical team** | Microvascular flap | 77 (100) | 100 (85-100) | 0 | 100 | 0.35 |
|  | LD flap | 45 (100) | 100 (80-100) | 34 | 100 |  |
|  | Fat graft | 17 (94) | 85 (70-100) | 30 | 100 |  |
|  | Implant | 6 (100) | 100 (80-100) | 80 | 100 |  |
|  | Overall | 145 (99) | 100 (80-100) | 0 | 100 |  |
| **Satisfaction with office**  **staff** | Microvascular flap | 76 (99) | 100 (77-100) | 0 | 100 | 0.20 |

| LD flap | 45 (100) | 100 (73-100) | 0 | 100 |
| --- | --- | --- | --- | --- |
| Fat graft | 17 (94) | 82 (63-100) | 17 | 100 |
| Implant | 6 (100) | 100 (76-100) | 73 | 100 |
| Overall | 144 (99) | 100 (73-100) | 0 | 100 |

LD, latissimus dorsi; N, number of patients

Supplemental Table 1e. Satisfaction with back, Physical well-being: Back and Shoulder, Satisfaction with abdomen and Physical well-being: Abdomen.

| **Scale** | **Reconstruction group** | **N (%)** | **Median (25th/75th**  **percentile)** | **Min** | **Max** |
| --- | --- | --- | --- | --- | --- |
| **Satisfaction with**  **back** | LD flap | 41 (91) | 66 (57-90) | 50 | 100 |
| **Physical well- being: back and**  **shoulder** | LD flap | 42 (93) | 61 (53-70) | 35 | 100 |
| **Satisfaction with**  **abdomen** | Abdominal flap | 58 (91) | 9 (8-10) | 3 | 12 |
| **Physical well-**  **being: abdomen** | Abdominal flap | 60 (94) | 65 (60-86) | 47 | 100 |

LD, latissimus dorsi; N, number of patients
